# Reticulon Homology Domain-Containing Proteins and ER-Phagy

**DOI:** 10.3389/fcell.2020.00090

**Published:** 2020-02-21

**Authors:** Manuela D’Eletto, Serafina Oliverio, Federica Di Sano

**Affiliations:** Department of Biology, University of Rome “Tor Vergata,” Rome, Italy

**Keywords:** autophagy, reticulon, membrane, endoplasmic reticulum, domain

## Abstract

The endoplasmic reticulum (ER) is a dynamic membrane system comprising different and interconnected subdomains. The ER structure changes in response to different stress conditions through the activation of a selective autophagic pathway called ER-phagy. This represents a quality control mechanism for ER turnover and component recycling. Several ER-resident proteins have been indicated as receptors for ER-phagy; among these, there are proteins characterized by the presence of a reticulon homology domain (RHD). RHD-containing proteins promote ER fragmentation by a mechanism that involves LC3 binding and lysosome delivery. Moreover, the presence of a correct RHD structure is closely related to their capability to regulate ER shape and morphology by curvature induction and membrane remodeling. Deregulation of the ER-selective autophagic pathway due to defects in proteins with RHD has been implicated in several human diseases, infectious and neurodegenerative diseases in particular, as well as in cancer development. While the molecular mechanisms and the physiological role of ER-phagy are not yet fully understood, it is quite clear that this process is involved in different cellular signaling pathways and has an impact in several human pathologies.

## Introduction

The endoplasmic reticulum (ER) is a wide and interconnected system of cell membranes forming a network with a common luminal space. It plays a central role in protein synthesis and modification, Ca^2+^ homeostasis, and lipid synthesis in eukaryotic cells (Chen et al., 2013). ER is a complex organelle composed of different dynamic subdomains, sheets, tubules, and a nuclear envelope, continuously remodeled to maintain cellular homeostasis (Schwarz and Blower, 2016). It is classified into smooth ER (SER) and rough ER (RER); the first is devoid of ribosomes and is characterized by a tubular structure, while the RER shows a sheet-like morphology and the presence of ribosomes (Chen et al., 2013).

The structure and function of ER are regulated by a variety of different proteins as well as by interactions with other organelles including mitochondria, Golgi, endosomes, lysosomes, peroxisomes, and the plasma membrane (Schwarz and Blower, 2016). Moreover, there is a close interaction with the cytoskeleton, which allows the ER tubules to elongate and to fuse (Gurel et al., 2014). The shape of ER changes in response to different cellular conditions and to specific signals. Moreover, the regulation of ER formation and morphology depends on different factors many of which have already been identified.

The structure of ER is also deeply affected by cellular stress, which induces ER remodeling; this is critical for cellular homeostasis and the prevention of various diseases (Hübner and Dikic, 2019). The dynamic changes of the ER depend on the activation of different pathways: ER-stress activated autophagy, which can induce ER expansion through the generation of membrane sheets (Schuck et al., 2009), or selective ER-phagy, which regulates ER turnover by a more specific mechanism (Grumati et al., 2018).

## Selective Autophagy

Autophagy is the major intracellular degradation pathway by which cytoplasmic materials are delivered to the lysosome and degraded. It represents a dynamic recycling mechanism essential for cellular homeostasis under basal and stress conditions. There are three different pathways of autophagy: macroautophagy, microautophagy, and chaperone-mediated autophagy (Ravikumar et al., 2010; Li et al., 2012; Cuervo and Wong, 2014).

Macroautophagy is characterized by the formation of an intermediate organelle called an “autophagosome,” a double-membraned vesicle containing the cargo material that sequesters a small portion of the cytoplasm and fuses with the lysosome allowing the degradation of cargos.

Although several proteins and the molecular mechanisms regulating autophagy have been characterized, the origin of the autophagosomal membrane is still poorly understood.

It has been proved that ER plays an important role in this process, providing not only the site for omegasome formation but also the membrane for the phagophore elongation (Nishimura et al., 2017; D’Eletto et al., 2019). On the basis of the cargo specificity and delivery mechanism, two different types of autophagy have been described: non-selective autophagy (microautophagy and macroautophagy), which implicates the digestion of cytoplasmic components in a relatively non-selective manner, and selective autophagy, which requires the recognition of autophagic substrates by specific receptors. The latter plays an important role in the targeting of specific organelles and cellular structures that are damaged or need to be turned over (Rogov et al., 2014). In the last few years, several mechanisms of selective autophagy have been characterized; these facilitate the targeted elimination of specific organelles via a receptor-mediated process and the cargo delivery into autophagosomes (Stolz et al., 2014).

In this context, recent studies have demonstrated that, under stress conditions caused by different stimuli (Moretti et al., 2017; Fregno and Molinari, 2018; Smith et al., 2018), ER fragments are eliminated by a distinct form of autophagy called ER-phagy, which regulates ER remodeling and represents an ER quality control mechanism (Grumati et al., 2018).

The word ER-phagy was first used by Walter et al. (Bernales et al., 2007) to describe ER-clearance in yeast, although the pathway had been previously defined in insects and mammalian cells (Fregno and Molinari, 2018). ER-phagy is a selective form of autophagy in which portions of the ER are sequestered within autophagosomal vesicles (AVs) and transported to the lysosomes for degradation. This process is mediated by specific ER-phagy receptors, ER-resident proteins containing an LC3/GABARAP-binding motif that allows their association with phagophore membrane and the recruitment of autophagy machinery to different ER regions (Grumati et al., 2018).

**FIGURE 1:**
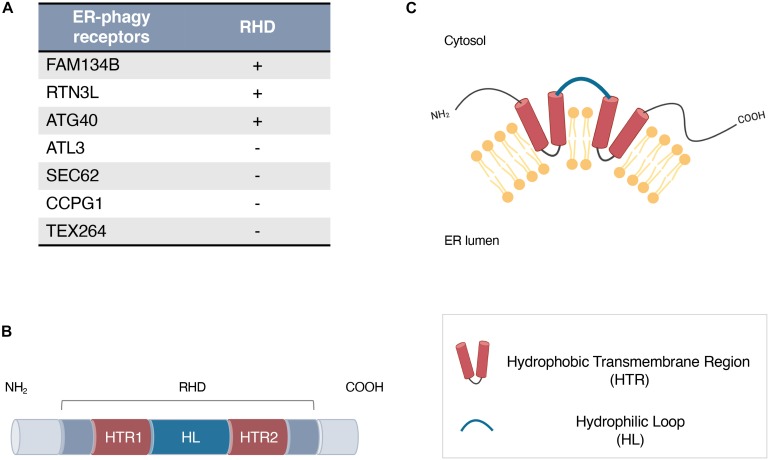
**(A)** ER-phagy receptors in mammals (FAM134B, SEC62, RTN3, CCPG1, ATL3, and TEX264) and in *Saccharomyces cerevisiae* (ATG40) harboring or not a Reticulon Homology Domain (RHD). **(B,C)** Schematic representation of the RHD structure **(B)** and topology **(C)**. An RHD consists of two large hydrophobic transmembrane segments (red, HTR1, HTR2), separated by a hydrophilic loop (blue, HL).

In mammals, six different receptors have been identified: FAM134B/RETREG1, SEC62, RTN3L, CCPG1, ATL3, and TEX264 (Khaminets et al., 2015; Fumagalli et al., 2016; Grumati et al., 2017; Smith et al., 2018; Chen et al., 2019; Chino et al., 2019). Some of them (FAM134B and RTN3L) are intramembrane proteins characterized by the presence of a specific domain called the reticulon homology domain (RHD), which is essential for their insertion in the ER membrane and ER-phagy regulation (Stolz and Grumati, 2019; Figure 1A).

## RHD-Containing Proteins

The RHD was originally identified as a distinguishing feature of two highly conserved protein families, reticulons (RTNs) and the functionally related REEPs/DP1/Yop1, both of which are classified as ER tubule-shaping proteins (Chen et al., 2013). Members of both families are expressed in all eukaryotes and localize predominantly to the tubular ER (Voeltz et al., 2006). These proteins do not hold any primary sequence homology but possess a conserved RHD region of ~200 amino acids consisting of two large hydrophobic transmembrane segments separated by a hydrophilic loop (Figure 1B). The hydrophobic segments form a hairpin within the lipid bilayer and act as a wedge structure, which is responsible for the retention and high membrane curvature stabilization of ER (Figure 1C; Voeltz et al., 2006). Overexpression of RTNs and DP1/Yop1 proteins generates long and unbranched tubules, while their depletion or elimination results in a significant reduction in ER tubules and abnormal ER morphology (Voeltz et al., 2006; Shibata et al., 2010).

In the mammalian genome, there are four independent RTN genes (RTN1-4) coding for different proteins ubiquitously expressed in vertebrates. Although each RTN possesses a variable N-terminal region, they all share a C-terminal RHD domain, which is also responsible for their ability to form homo and heteromers and allows organization in a scaffold on the tubular ER (Di Sano et al., 2012). It has been demonstrated that RTNs bind to atlastins, evolutionarily conserved dynamin-like GTPases, and co-operate to form the network of interconnected ER tubules (Hu et al., 2009).

The REEP family includes six members, REEP1–6, which are orthologs of yeast Yop1 in mammals. REEPs also regulate the tubular ER network in co-operation with atlastins (Park et al., 2010).

More recently, a RHD has been identified in other proteins that do not belong to RTNs or REEPs/DP1/Yop1 families. Interestingly, among these, there are different ER-phagy intramembrane receptors: ATG40 in *Saccharomyces cerevisiae* and FAM134B in mammals (Figure 1A; Khaminets et al., 2015; Mochida et al., 2015). The role of these proteins in ER-phagy regulation is strictly related to the presence of LC3-interacting regions (LIR), by which they are able to bind to LC3/GABARAP and to recruit the autophagy machinery to different ER regions (Grumati et al., 2018). Nevertheless, it has been demonstrated that a correct ER-phagy flux strongly depends on the presence and structural integrity of their RHD (Grumati et al., 2018).

## RHD Structure and Autophagy

FAM134B was the first ER-phagy receptor to be identified and characterized (Khaminets et al., 2015). It is an intra-membrane ER-resident protein that is mainly localized to the edges of the ER sheets and is a member of the FAM134 family.

FAM134B is able to bind LC3 on forming autophagosomal membranes via an LIR and subsequently to address fragmented ER sheets to lysosomes. This is essential for the control of ER morphology and turnover, as confirmed by modulation studies of FAM134B expression. In fact, it has been reported that there is an expansion of the ER when the protein is downregulated, while FAM134B overexpression results in ER fragmentation and lysosomal degradation (Khaminets et al., 2015).

Interestingly, the capability of FAM134B to sense and induce membrane curvature during ER-phagy is strictly dependent on its RHD structure and topology, which are fundamental for the capability of the protein to regulate membrane remodeling and ER degradation. This notion is supported by the fact that RHD disruption affects selective ER-phagy flux and is associated with pathological states (Bhaskara et al., 2019).

The relevance of the RHD in the regulation of ER-phagy processes is also supported by the fact that other selective ER-phagy receptors harbor the same conserved region. For example, in *S. cerevisiae*, an RHD domain has been identified in the autophagic receptor Atg40, an essential protein for ER-phagy induction, as demonstrated by knockout studies that completely blocked this pathway (Mochida et al., 2015). Atg40 largely colocalizes with Rtn1 to ER tubules and the edges of ER sheets and, consistent with its localization, degrades cortical ER (Chen et al., 2018).

RTN3, a member of the RTN family, is an RHD-containing protein found at ER tubules and implicated in their specific turnover after autophagic induction (Grumati et al., 2017). As a member of the RTN family, RTN3 has previously been shown to be involved in the formation of ER tubules. The longest isoform of RTN3 contains six active LIR domains in its N-terminal region, which are crucial for LC3/GABARAP binding, fragmentation of ER tubules, and its function as an ER-phagy receptor (Grumati et al., 2017). The ablation of Atg40, FAM134B, and RTN3 does not affect the general macro-autophagy flux, but similarly, the specific role of the above-mentioned receptors, which are active under specific conditions to regulate ER-phagy, is functionally related to their RHD.

Recently, a role in the regulation of autophagic processes has been demonstrated for another member of the RTN family, RTN-1C (D’Eletto et al., 2019), supporting the notion that RHD-containing proteins represent important molecules in the control of autophagic mechanisms. However, since various studies have reported a crucial role in the induction of ER phagy for proteins that do not possess an RHD domain (Fumagalli et al., 2016; Smith et al., 2018; An et al., 2019; Chino et al., 2019), further insights are needed in order to clarify the importance of this specific domain and the different molecular mechanisms regulating the process.

## Selective Autophagy and Disease

Several studies have underlined the importance of selective autophagy in human diseases. On the basis of the selectivity of clearance, autophagy has been linked to a wide range of human diseases, including cancer, chronic liver disorders, pulmonary diseases, and neurodegenerative diseases. In particular, dynamic remodeling of the ER is fundamental for cellular homeostasis and the prevention of disease pathogenesis. In fact, a defect in correct ER shaping or remodeling and ER homeostasis is dangerous to cells and seems to be common to several human diseases, including infectious, neurodegenerative diseases, and cancer.

Accordingly, several studies have demonstrated that different ER-phagy receptors are linked with various human disorders (Table 1). For example, mutations in FAM134B and ATL-3 have been shown to affect the survival of sensory and autonomic neurons, leading to hereditary sensory neuropathies (HSAN) (Kurth et al., 2009; Kornak et al., 2014). In some patients, leg spasticity and weakness have also been reported (Eggermann et al., 2018), suggesting that axons of upper motoneurons can be affected as well. Nevertheless, the molecular mechanism linking these proteins to HSAN pathology is only partially understood.

**TABLE 1:** Diseases correlated to mutation in different ER-phagy receptors.

	**FAM134B**	**RTN3L**	**ATL3**	**SEC62**	**CCPG1**
Monogenic disorders	HSAN2B		HSN1F		
	Esophageal squamous carcinoma	Alzheimer’s disease		Non-small cell lung cancer	Pancreatic cancers
Related diseases	Colon and Breast cancer	Infectious diseases		Prostate and thyroid cancer	
	Infectious diseases			Squamous cell carcinoma	

*Monogenic disorders are caused by mutation of the indicated genes, while related diseases are associated with up- or down-regulation of the respective gene.*

The relevance of ER-phagy on neuronal homeostasis is further suggested by RTN3 involvement in the etiology of neurodegenerative Alzheimer’s disease. It has been observed that RTN3 deficiency is correlated with an increase of amyloid deposition in mouse models of the disease (Shi et al., 2014; Zou et al., 2018). Moreover, RTN3 variants have been identified in patients with sporadic early- and late-onset Alzheimer’s disease (Zou et al., 2018), even if a direct correlation with the role of RTN3 in ER-phagy remains to be determined.

Endoplasmic reticulum-phagy is considered an important defense mechanism to eliminate viruses or bacteria that use ER compartments during the infection cycle. Both FAM134B and RTN3 have been associated with the control of virus replication (Wu et al., 2014; Chiramel et al., 2016).

Interestingly, in some cases, the loss of function of the ER-phagy receptor is a consequence of RHD destruction by a specific virus protease (Lennemann and Coyne, 2017).

Finally, recent findings have suggested that ER-phagy-related receptors are associated with several types of cancer, although a different role for these proteins has been observed in the pathogenesis of different tumors. FAM134B is implicated in esophageal squamous carcinoma as a cancer development oncogene and, by contrast, as a tumor suppressor in colon and breast cancer (Tang et al., 2007; Islam et al., 2017). SEC62 upregulation was reported in non-small cell lung cancer and prostate and thyroid cancers as well as different carcinomas (Linxweiler et al., 2012; Bergmann et al., 2017).

It has been suggested that enhanced ER-phagy via SEC62 may render the tumor cells more resistant to ER stress (Bergmann et al., 2017).

Altogether this evidence highlights the emerging relevance of ER-phagy pathways and receptor proteins in several human diseases. Moreover, an important role of proteins containing an RHD domain is also supported by the fact that mutations of these membrane-shaping proteins are implicated in different human disorders (Hübner and Kurth, 2014), suggesting their putative involvement in the ER-phagy mechanism.

## Conclusion

The importance of selective autophagy in the pathogenesis of different human disorders, together with the characterization of its physiological and pathophysiological role, are certainly very interesting and promising fields of investigation. In particular, there is increasing interest in and research on the major challenging topic of ER-phagy, which will provide a better understanding of this process in order to assess whether targeting ER-phagy may be considered an effective therapeutic strategy.

Of course, further insights and more focused studies are needed to define the molecular pathways responsible for activation of ER-phagy; for example, it will be interesting to evaluate the importance of the different ER-phagy receptors and the regulation of their expression levels. Moreover, it will be relevant to achieve a better characterization of the role of the conserved receptor structure (i.e., the presence of RHD) and how this may affect membrane remodeling and fragmentation during ER-phagy.

Hopefully, deeper analysis of the different proteins participating in ER-phagy regulation may further improve our current knowledge on the full potential of these molecules, not only as functional participants in pathophysiological events but as pharmacological targets for different human diseases.

## Author Contributions

All authors drafted the manuscript. MD’E generated the illustration. FD and MD’E wrote the manuscript.

## Conflict of Interest

The authors declare that the research was conducted in the absence of any commercial or financial relationships that could be construed as a potential conflict of interest.
